# Help-seeking patterns and level of care for individuals with bipolar disorder in Rwanda

**DOI:** 10.1371/journal.pgph.0002459

**Published:** 2023-10-10

**Authors:** Caroline Juhl Arnbjerg, Emmanuel Musoni-Rwililiza, Nelly Umulisa Rurangwa, Maja Grønlund Bendtsen, Chantal Murekatete, Darius Gishoma, Jessica Carlsson, Per Kallestrup

**Affiliations:** 1 College of Medicine and Health Sciences University of Rwanda, Kigali, Rwanda; 2 Center for Global Health, Department of Public Health, Aarhus University, Aarhus, Denmark; 3 Mental Health Department, University Teaching Hospital of Kigali, Kigali, Rwanda; 4 Competence Centre for Transcultural Psychiatry (CTP), Mental Health Centre Ballerup, Ballerup, Denmark; 5 Department of Clinical Medicine, University of Copenhagen, Copenhagen, Denmark; University of the Witwatersrand, SOUTH AFRICA

## Abstract

Most descriptive data on individuals with bipolar disorder originate from high-resource settings. Very little is known about the accessibility and service provision of intensive mental health care to persons living with bipolar disorder in low-resource settings. This information is needed to inform health systems and guide practitioners to improve standard treatment options and access to treatment. This cross-sectional study explored the level of care for outpatients with bipolar disorder and their help-seeking patterns at the two national referral hospitals in Rwanda. The study found that the majority, 93%, of outpatients with bipolar disorder in Rwanda were on prophylactic psychopharmacological treatment, but mainly first-generation antipsychotics and just 3% received lithium treatment. Furthermore, there was a lack of psychosocial intervention; consequently, 44% were not aware that they had bipolar disorder. Moreover, 1 in 5 participants utilized or had previously used traditional medicine. Awareness of own diagnostic status was not associated with educational level or use of traditional medicine. The study’s sample size of 154 patients is relatively small, and the cross-sectional design does not provide causal inferences. The results demonstrate a considerable unmet need for improved mental health care services for individuals with bipolar disorder in Rwanda, including access to optimal medication and psychosocial interventions. Psychoeducation could be a possible starting point for improving the standard of care, informing the individual on their diagnosis and medication while empowering them to engage in their treatment plan.

**Trial registration**: ClinicalTrials.gov NCT04671225. Registered on November 2020.

## Introduction

Bipolar disorder (BD) is a severe mental health disorder characterized by episodes of mania, hypomania, depression, and mixed states [1]. The lifetime prevalence of BD in the general population is estimated to be around 1–2%, with some studies suggesting rates as high as 4% [2–4].

Individuals with BD have one of the highest rates of suicide among persons with psychiatric conditions, approximately 20–30 times higher than that of the general population [5–7]. Early detection of BD is challenging, patients are often misdiagnosed, and treatment is often delayed [8, 9]. The treatment delay is associated with a further elevated risk of suicide and poorer functional outcomes [10]. Although BD is not curable, a combination of medications, psychotherapy, and lifestyle changes can effectively manage symptoms, prevent relapse and improve the overall quality of life for individuals with BD [11]. However, the global treatment gap for severe mental disorders, including BD, is substantial—particularly with regard to low- and middle-income countries (LMICs), where around 85% of the world’s population resides [12]. Estimates show that around four out of five individuals with severe mental disorders in LMICs did not receive care in a given year [13]. In addition, a study from Ethiopia conducted in 2019 found that 72% of those who received biomedical care for psychosis did not receive minimally adequate treatment, defined as at least four monitoring visits per year [14].

One of the significant barriers to decreasing the treatment gap in LMICs is the lack of human resources. This may explain why a significant proportion, up to 50%, of African individuals seeking mental health care resort to traditional and religious healers [15].

Treating BD in low-resource settings is further complicated by the limited local availability of appropriate medication, such as mood stabilizers and atypical antipsychotic drugs, and the challenge of using these drugs safely in those settings [16]. In addition, there is a severe lack of research on both acute and prophylactic interventions for individuals with BD in low-resource settings [17]. Data on the available care provision and the individuals’ pathways to care is needed to develop strategies and contextualized interventions for improving the treatment for individuals with BD in low-resource settings.

In Rwanda, a Sub-Saharan country with approximately 14 million citizens and just 15 psychiatrists in 2023, access to formal mental health services often begins at health centers. From there, one may be referred to district hospitals and, if needed, referred to specialized care at the national referral hospitals. In cases of self-payment or involuntary hospitalizations, direct access to service at referral hospitals is also possible.

This study was conducted to shed light on help-seeking patterns and levels of insight into own illness among patients with BD in Rwanda.

## Methods

### Study design and settings

The present study is a cross-sectional investigation of baseline data from a randomized clinical trial (RCT) on psychoeducation for persons with BD in Rwanda. Further details on the RCT are presented in the trial protocol [18].

Participants were enrolled in the study from January 2021 through March 2021. They were recruited from either one of the only two established referral hospitals with mental health treatment capabilities in the capital city of Rwanda, Kigali: CARAES-Ndera Hospital and The University Teaching Hospital of Kigali (CHUK). CARAES-Ndera Hospital is a mission health facility, yet the government of Rwanda supports the hospital by providing human resources to the hospital and assisting in its management, and the singular neuropsychiatric hospital in the country with inpatient care that offers specialized healthcare in psychiatry and neurology. The hospital is located 17 kilometers from Kigali City, while CHUK is the largest referral hospital in Rwanda, located in Kigali City. This hospital does not provide inpatient care for psychiatric patients, despite it being the second biggest outpatients’ mental health clinic.

The study is reported in accordance with the Strengthening the Reporting of Observational Studies in Epidemiology (STROBE) guidelines [19] (S1 Checklist).

### Study population and sampling

Outpatient adults, 18 years or older, diagnosed with bipolar disorder type I or II in a current euthymic state, were included in the study.

Persons with mental retardation documented in their medical records or currently active alcohol- or drug-use disorders, a diagnosis of deafness (because of various tests that need verbal interactions), and those who declined to give consent or had previously participated in any structured psychological intervention were excluded from the study. However, following the completion of the study, persons diagnosed with deafness will be offered the study material and invited to future group psychoeducation sessions conducted outside the study, with the possibility of a sign language translator participating.

The clinical staff reviewed medical records and reached out to outpatients diagnosed with BD, inviting them to visit the hospital facility for further information about the project. Those who expressed interest underwent an eligibility assessment and received an invitation to participate in the study if deemed eligible. Patients living in the province of Kigali were prioritized due to the inter-district travel restrictions that the Government of Rwanda imposed to ensure the safety of the citizens of Rwanda in response to the surge in COVID cases at the time of enrollment.

The estimated sample size for the RCT was determined to be 50 participants per arm, totaling 100 participants, taking into account a relapse incidence of 92% in the control group compared to 67% in the psychoeducation group over a two-year period. This sample size was calculated to achieve 80% power with a significance level of 5% while also accounting for a dropout rate of 20% [18, 20].

### Clinical assessment and study tools

A trained psychiatrist used The Mini-International Neuropsychiatric Interview (MINI) to confirm the psychiatric diagnosis. Past traumatic events were recorded using Life Event Checklist for DSM-5 (LEC-5).

In collaboration with the clinical staff, the participants filled out demographic information regarding their living conditions, past psychiatric history, and their use of mental health service data. This information was not available in the medical records. In addition, information on the type of health insurance and poverty level categories (Ubudehe) was collected. Rwanda follows a universal healthcare model, providing health insurance through the Mutuelles de Santé program. Members pay less than a dollar to visit health centers or 10 percent of the total bill at all districts and referral hospitals, including the cost of prescribed medication from the National List of Essential Medicines. The cost of health insurance is determined by the poverty level categories assigned to the individuals. These categories range from A to E and reflect different levels of house income. Categories A and B comprise households that are self-reliant, while C and D indicate partial dependency on social protective schemes. Category E encompasses individuals who benefit from full state social protection and are not expected to transition out of this level [21, 22].

Before the initiation of data collection, the clinical staff underwent training in applying the Hamilton Depression Scale-17 (HDRS-17) and the Young Mania Rating Scale (YMRS). Both scales were used to assess the current clinical mood state of the participants before inclusion and to ensure that the participant was in an euthymic state, the latter defined as a score of 16 or less on the HDRS-17 and a score of 11 or less on the YMRS.

The MINI, LEC-5, and HDRS-17 are validated in Kinyarwanda, while the YMRS is currently undergoing validation.

### Ethical considerations

The study was conducted in accordance with the Declaration of Helsinki and approved by the University of Rwanda College of Medicine and Health Sciences Institutional Ethical Review Board (No 049/CMHS IRB/2020) and the ethical review boards at CARAES-Ndera Hospital and CHUK. In addition, the study investigators obtained approval to conduct research in Rwanda from the National Council for Sciences and Technology, as is required for all researchers in Rwanda. All study participants gave written informed consent for study participation. The information of all the participants was kept anonymous, and confidentiality was properly maintained. The first author had access to information that could identify individual participants during or after data collection. The protocol is registered on ClinicalTrials.gov ID NCT04671225, November 2020.

### Statistical analysis

Participants’ backgrounds and clinical characteristics were summarized as the frequency and percentages for categorical variables and the median and interquartile range (IQR) for continuous variables. Continuous data were compared using the Student’s t-test or Mann–Whitney U test. The chi-square test was utilized to test for associations between awareness of own illness and categorical background or clinical data. A p-value <0.05 was considered statistically significant throughout the analysis. All analyses were performed using STATA version 17.0 (StataCorp. Texas, USA).

## Results

**Fig 1:**
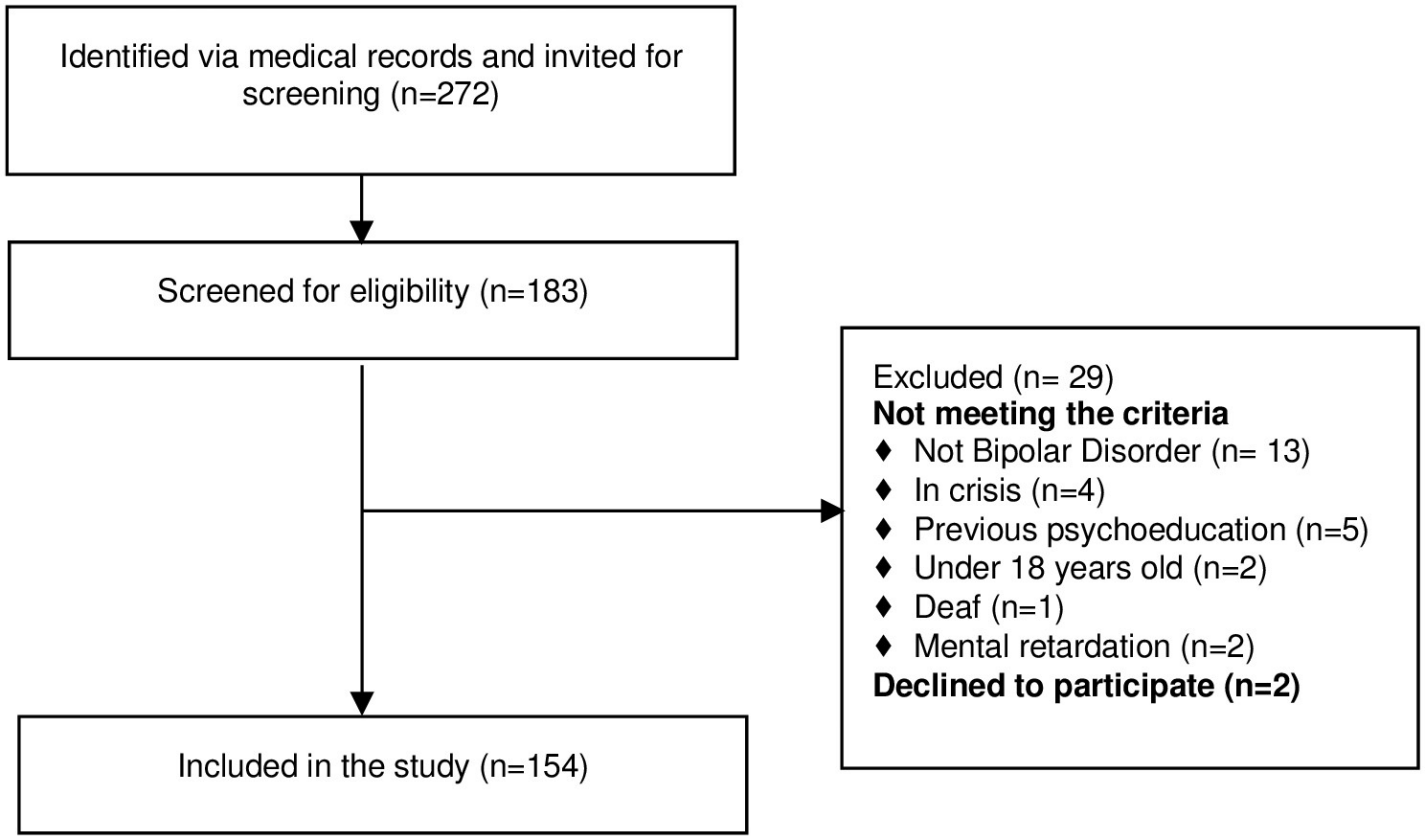
Flow diagram for inclusion and exclusion.

A total of 154 persons with BD were enrolled in the study. Fig 1 details the participant enrollment flow diagram.

Table 1 summarizes the sociodemographic characteristics. The sample consisted of an equal proportion of men and women, with a mean age of 35.7 years and the majority having a diagnosis of BD type I (80%). About 30% had no formal education or an educational level equal to primary school, and almost 2 out of 3 were unemployed. The median monthly household income was 55 USD; just 1 in 4 was from social protection schemes (poverty levels A and B) [21].

**Table 1:** Summary of the sociodemographic characteristics of the sample.

Age, years	35.7 (9.4)
Female sex, % (n)	50 (77)
Educational level, % (n)	
No formal education	6 (10)
Primary school	25 (39)
Lower secondary school	16 (24)
Upper secondary school	27 (42)
Vocational studies	6 (9)
University Degree	19 (30)
Literacy rate, % (n)	94 (145)
Civil status, % (n)	
Single	54 (83)
Married	35 (54)
Divorced	8 (13)
Widowed	3 (4)
Occupational status, % (n)	
Working	26 (40)
Unemployed	62 (96)
Retired	2 (3)
Student	10 (15)
Poverty levels (Ubudehe), % (n)	
A	6 (9)
B	17 (26)
C	43 (66)
D	21 (33)
E	13 (20)
Insurance, % (n)	
Governmental	97 (149)
Private	3 (5)
Monthly household income in USD (n = 126)	55 [18, 185]
Religious, % (n)	94 (144)

(N = 154, except where noted)

Data are mean (S.D.), median [IQR], or % (n) unless otherwise stated.

The clinical characteristics of the sample are presented in Table 2. The average age at illness onset was 23.3 years, and the average illness duration was 11 years. Eleven percent fulfilled the criteria for PTSD, yet when screened for potentially singular traumatic events in the participant’s lifetime using the Life Events Checklist for DSM-5 (LEC-5), the greater majority of participants had experienced multiple traumatic events meeting the criteria. Three out of four reported that severe human suffering had “happened to me” or they had “witnessed it,” 70% had experienced physical assaults, and 45% had been in active combat or exposure to a war zone. HIV was the most common somatic comorbidity, with 9% of the study population reporting an HIV diagnosis.

**Table 2:** Summary of the clinical characteristics of the sample.

Clinical data	
Bipolar subtype, % (n)	
Bipolar Disorder I	80 (123)
Bipolar Disorder II	20 (31)
Age at onset, years	23.3 (7.6)
Illness duration, years	11 [5–17]
**Traumatic life-events (LEC-5), % (n)** ^a^	
Natural disaster	39 (53)
Fire or explosion	39 (53)
Transportation accident	60 (82)
Serious accident at work, home or during recreational activity	30 (41)
Exposure to toxic substance	14 (19)
Physical assault	70 (96)
Assault with a weapon	40 (55)
Sexual assault	34 (46)
Other unwanted or uncomfortable sexual experience	36 (49)
Combat or exposure to a war zone	45 (61)
Captivity	28 (39)
Life-threatening illness or injury	55 (76)
Severe human suffering	75 (103)
Sudden violent death	42 (58)
Sudden accidental death	41 (56)
Serious injury, harm, or death respondent caused to someone else	32 (44)
Any other very stressful event or experience	71 (97)
PTSD diagnosis, % (n)^a^	11 (14)
Somatic comorbidity, % (n)	19 (29)
HIV, % (n)	9 (14)
Affective episodes irrespective of polarity, % (n)	
1 to 5	68 (106)
6 to 10	11 (17)
Above 10	21 (31)
Previous psychiatric hospitalization, % (n)	95 (146)
No. of prior psychiatric hospitalizations^b^	3 [2–6]
Suicidal behavior, % (n)	
Previous suicidal attempt	32 (49)
Family history of psychiatric disorder, % (n)	
No history	39 (60)
First degree relative with psychiatric disorder	28 (43)
Do not know	33 (51)

(N = 154, except where noted)

Data are mean (S.D.), median [IQR], or % (n) unless otherwise stated.

^a^Only traumatic events experienced or witnessed by the respondent are shown in Table 2. Seventeen participants declined to fill out the LEC-5, and 12 participants did not complete the MINI interview chapter H.

^b^Among the 146 participants who had previously been hospitalized

Regarding the severity of the BD, one-third of all the participants had a history of previous suicidal attempts with no risk difference between BD subtype I and subtype II. Ninety-five percent had previously been hospitalized due to their illness, with a median number of three psychiatric hospitalizations over the current course of their illness.

All participants included were in remission, with a median duration of 52 weeks (Table 3). In the measurement of the internalized stigma, the majority report either minimal (30%) or mild internalized stigma (51%), and 1 percent have a score of severe internalized stigma.

**Table 3:** Clinical rating scales.

Current clinical status	
HAM-D	4.7 (4.7)
YMRS	2.1 (3.4)
Time in remission, weeks	52 [16–156]
**Internalized-Stigma**	
ISMI-10^c^, % (n)	
Minimal to no internalized stigma	30 (41)
Mild internalized stigma	51 (69)
Moderate internalized stigma	17 (23)
Severe internalized stigma	1 (2)

Data are mean (S.D.), median [IQR], or % (n) unless otherwise stated.

^c^Nine-teen participants did not report in ISMI-10.

### Awareness, help-seeking behavior, and treatment received

Table 4 shows the participants’ awareness of their own diagnosis, pathways to treatment, and the use of medical treatment and alternative treatment among the participants.

**Table 4:** Awareness, help-seeking behavior, and treatment received.

Awareness of own diagnostic status Knowledge on own disease	
Unknown diagnostic status, % (n)	44 (68)
**Help-seeking behavior**	
Who did you first seek for support, % (n)	
Family	80 (123)
Friends	9 (14)
Others	11 (17)
Who initiated the first contact to professional help services, % (n)	
The person themselves	8 (13)
Relatives/friends	80 (123)
Colleagues	3 (4)
The Police	3 (4)
Others	6 (10)
**Psychopharmacological treatment, % (n)**	
Undergoing psychopharmacological treatment,	93 (142)
Antipsychotics,	81 (125)
First generation	90 (112)
Second generation	20 (25)
Anticonvulsants	59 (91)
Antidepressants	4 (6)
Lithium	3 (4)
**Alternative treatment, % (n)**	
Have visited a religious leader because of bipolar disorder	51 (78)
Have visited a traditional healer because of bipolar disorder	31 (47)
Have used/is using traditional medicine	23 (36)

Data are mean (S.D.) or % (n) unless otherwise stated.

The listed anticonvulsants were all prescribed for the treatment of BD.

When asked if a health professional ever told them that they have BD or manic-depressive illness, 44% answered no and said they did not know they had a BD diagnosis at enrollment. Many respondents did not know that what they experienced had a term, or they thought they had a diagnosis of schizophrenia.

When asked about help-seeking behavior, four out of five reported that they first sought help from their families and that family members or friends initiated the first contact with professional treatment services.

Ninety-three percent of the participants enrolled reported receiving psychopharmacological treatment, of which 81% received antipsychotics, 60% used anticonvulsants, and 3% received lithium. Of participants receiving antipsychotics, the greater majority equating to 90%, were on first-generation antipsychotics, and only 20% were on second-generation antipsychotics. Both valproate and carbamazepine were administered to a similar extent in the treatment of participants, while lamotrigine was not prescribed to any of the enrolled participants. Of the 42 participants receiving valproate, only 11 were females of reproductive age (data not listed in tables).

Exploring the use of alternative treatment, half of the study population reported that they had visited a religious leader because of their BD, and around one-third had visited a traditional healer. Twenty-three percent have or are still using traditional medicine for their BD. None of the participants had received any structured psychosocial intervention as it was an exclusion criterion. Yet, when assessing how many were excluded during enrollment for that reason, only five out of 170 with BD had received any structured psychosocial intervention (Fig 1).

### Factors associated with knowledge of own diagnostic status in people living with BD in Rwanda

Participants who had previously attempted suicide were more likely to have been informed of their BD diagnosis compared to those without a history of suicidal attempts (67% vs. 50%, p = 0.05). Likewise, participants with BD subtype II were more likely to be aware of their diagnosis than those with subtype I (77% vs. 50%, p = 0.007). The number of hospitalizations and episodes or use of alternative treatment was not significantly associated with awareness of their own diagnostic status. Neither were educational levels or illness duration. No significant differences were observed in these parameters between participants with BD subtype I and subtype II or between participants with and without a history of suicidal attempts.

## Discussion

The study was conducted to assess the awareness of own illness, the help-seeking patterns, and level of care for outpatients with BD in Rwanda to guide the development of future prophylactic interventions tailored to low-resource settings. The most unanticipated finding from this study was that nearly half of the outpatients with BD, 44%, receiving care at tertiary hospitals in Rwanda did not know what psychiatric diagnosis they received treatment for. This finding was not associated with the level of education. Almost all were on prophylactic psychopharmacological treatment, mainly first-generation antipsychotics and anticonvulsants, with very few, three percent, currently receiving lithium therapy. Only eight percent had them-self initiated the first contact with professional help, one in three had visited a traditional healer because of BD, and one in four were using or had previously used traditional treatment modalities.

In line with the findings from this study, a previous study from South Africa in 2015 on inpatients’ awareness of their clinical condition found that roughly half of the respondents knew the possible causes of their condition, and three-quarters knew their diagnosis, regardless of respondents’ educational level [23]. The study mentions time constraints as one reason for the failure to provide patients with information. Awareness and knowledge of one’s own diagnosis and the capacity to use that knowledge effectively is a fundamental aspect of self-empowerment. This, in turn, empowers patients to take an active role in managing and improving one’s health [24]. A recent systematic review and meta-analysis from 2021 on the effectiveness of adjunctive psychotherapy for BD concluded that healthcare systems should offer a combination of pharmacotherapy and psychotherapy, such as family or group psychoeducational therapy for outpatients with BD [25]. In contrast, a review from 2018 found that no study on psychosocial intervention for individuals with BD has been conducted in a low-income country [26]. In addition, it needs to be explored if interventions for BD, such as psychoeducation, can be decentralized, considering the current ratio of 1.4 mental health workers per 100.000 individuals in Africa and one psychiatrist per one million citizens in Rwanda [27, 28]. One first step in decentralizing care could be delivering psychosocial support at district hospitals, leveraging the existing workforce of 600 psychologists and 500 mental health nurses working in the country’s healthcare sector. Moreover, at the community level, support groups facilitated by lay counselors, including CHWs with specialist support, could empower the individual and the community surrounding persons with BD, encompassing their families, friends, and the broader society. It has been suggested that family support plays a much larger role in the recovery from severe mental health illness in LMICs compared to high-income settings, and social connectedness and interdependence may be more pertinent indicators of recovery than independence and autonomy [29]. Moreover, this present study emphasizes the importance of incorporating elements of spirituality and traditional medicine into the psychoeducational information provided to participants, given that patients are utilizing them as concurrent traditional treatment modalities. Important to note; the use of traditional healers and medicine was not negatively associated with awareness of own diagnostic status. It is possible that the use of religious leaders and traditional healers is a result of a lack of human resources in the professional mental health care system.

In our study, the prevalence of previous suicidal attempts was 32%, similar to global estimates [30], and individuals with a history of prior suicide attempts were more likely to have been informed about their BD than those who had no previous suicidal attempts. This finding supports the notion of an overburdened healthcare system where mental health workers do not have the time and resources to inform individuals about their condition, except those in severe distress or direct life-threatening danger. No difference between the participants with BD subtype I and II was found in the data to explain why participants with BD subtype were more likely to know their diagnostic status. Yet, one explanation could be that BD subtype II is especially hard to diagnose accurately because of the difficulty in differentiating it from recurrent unipolar depression [31]. The diagnostic process for bipolar II may require more time for the clinician to engage in thorough conversations with the patient, potentially leading to a higher likelihood of the patient receiving more information about their clinical condition.

The WHO Mental Health Gap Action Programme (MhGAP), aiming at scaling up mental health services in LMICs, recommends lithium or valproate for the maintenance treatment of manic episodes in BD unless the person is female of conceptive age; then valproate should be avoided because of risk of teratogenicity. If these options are not feasible, then haloperidol, chlorpromazine, or carbamazepine may be used as alternative medical treatment approaches [32, 33]. Overall the prescription patterns identified in this study align with these recommendations, except lithium not being available and valproate being prescribed to 11 women of reproductive age. It is evident that women of reproductive age face additional challenges in accessing effective prophylactic treatment for BD when lithium treatment is not available. This calls for an investment in affordable and safe monitoring options for lithium administration. As for now, the only governmental healthcare facility in Rwanda that has the capacity to do regular lithium level toxicity screening is CARAES- Ndera Hospital, but the cost is high for the patient.

The median monthly household income among the study participants was only 55 USD, making it financially challenging for most to afford out-of-pocket medical expenses. Currently, due to their high cost, either directly or in administration, most second-generation antipsychotics and lithium are not included in the National List of Essential Medicines for Adults in Rwanda. They are, therefore, not covered by governmental insurance. Combined with the risk of intermittent shortages further limits access to optimal medical therapeutic regiments [34].

In addition, almost one in 10 in the study had HIV, compared to a prevalence of 3% in the general population of Rwanda. These findings further reinforce the vulnerability of individuals with BD to HIV, particularly in sub-Saharan Africa, the region most heavily impacted by the HIV epidemic globally [35, 36].

The study also revealed that a substantial number of the participants reported experiencing a high number of traumatic events, yet only 11% had a comorbid PTSD diagnosis. The most commonly reported traumatic events among the participants were severe human suffering and physical assaults, with a staggering 70% of individuals having experienced these events. It is well known that childhood traumatic events are risk factors for developing BD and, in addition, a more severe clinical presentation over time, including an increased risk of suicide attempts [37]. Given the devastating history of the country with the Genocide Against the Tutsi in 1994, one may assume the prevalence of BD in Rwanda to be high [38]. Yet, according to the first Rwandan National Mental Health Survey conducted in 2018, the prevalence of BD is estimated to be only 0.7% [39]. Globally, there is no clear evidence for differing prevalence rates across ethnic groups, and cultural factors and lack of human resources may explain the low reported prevalence in Rwanda, indicating a possible greater number of undetected cases [40]. The present study stresses the importance of support from family and close friends when it comes to the individual with BD getting professional help.

Due to the relatively high proportion of participants without awareness of their own diagnostic status of BD, it was not possible to estimate the diagnostic delay for the participants. The same applies to an intended exploration of the genetic component of BD in our sample, as one-third of the participants did not know their family history. In addition, the cross-sectional study design is a limitation, as it prevents any causal relationship analysis. Finally, it is important to note that the participants in this study were selected from outpatient clinics at tertiary hospitals and mainly residing in the capital city, and almost all had a history of hospitalization at the only mental health hospital in Rwanda. Therefore, the findings of this study may only represent a small subset of individuals with BD in Rwanda who have access to specialist care in urban settings.

## Conclusion

In conclusion, outpatients with BD in Rwanda at the tertiary level receive some degree of prophylactic psychopharmacological treatment, but mainly first-generation antipsychotics, and lack access to lithium treatment. The study further highlights the add-on challenges for women of reproductive age in accessing effective prophylactic treatment. Detailed in the study is also a severe lack of awareness of own diagnostic status of BD irrespective of educational level, as roughly half of the outpatients report that they have never been informed that they have BD. Implementation and research focusing on adjunctive psychoeducation informing and empowering individuals with BD to take an active role in their treatment is a crucial step towards improving the care for individuals with BD in a low-resource setting, and our study demonstrates that these health gaps exist to this day in Rwanda.

## Supporting information

S1 Checklist STROBE checklist. (DOCX)
